# Fused Heterocyclic Systems with an s-Triazine Ring. 34. Development of a Practical Approach for the Synthesis of 5-Aza-isoguanines

**DOI:** 10.3390/molecules24081453

**Published:** 2019-04-12

**Authors:** Ahmad Junaid, Felicia Phei Lin Lim, Yvonne Peijun Zhou, Wai Keung Chui, Anton V. Dolzhenko

**Affiliations:** 1 School of Pharmacy, Monash University Malaysia, Jalan Lagoon Selatan, Bandar Sunway, Selangor Darul Ehsan 47500, Malaysia; ahmad.junaid@monash.edu (A.J.); felicia.lim@monash.edu (F.P.L.L.); 2 Department of Pharmacy, Faculty of Science, National University of Singapore, 18 Science Drive 4, Singapore 117543, Singapore; marz_vonne@yahoo.com.sg (Y.P.Z.); phacwk@nus.edu.sg (W.K.C.); 3 School of Pharmacy and Biomedical Sciences, Curtin Health Innovation Research Institute, Faculty of Health Sciences, Curtin University, GPO Box U1987, Perth, WA 6845, Australia

**Keywords:** triazine, triazole, azapurine, purine isostere

## Abstract

Purine isosteres present excellent opportunities in drug design and development. Using isosteres of natural purines as scaffolds for the construction of new therapeutic agents has been a valid strategy of medicinal chemistry. Inspired by the similarity to isoguanine, we attempted to develop a practical method for the preparation of 5-aza-isoguanines. Several synthetic approaches were explored to establish a robust general protocol for the preparation of these compounds. The significant difference in the reactivity of the C-5 and C-7 electrophilic centers of 1,2,4-triazolo[1,5-*a*][1,3,5]triazines (5-azapurines) towards nucleophiles was demonstrated. The most practical and general method for the preparation of 5-aza-isoguanines involved a regioselective reaction of ethoxycarbonyl isothiocyanate with a 5-aminotriazole. The intramolecular ring closure of the resulted product followed by the S-methylation afforded 7-methylthio-2-phenyl-1,2,4-triazolo[1,5-*a*][1,3,5]triazin-5-one, which could be effectively aminated with various amines. The resulted 5-aza-isoguanines resemble a known purine nucleoside phosphorylase inhibitor and could be interesting for further investigations as potential anticancer agents.

## 1. Introduction

Purine isosteres attract significant attention from medicinal chemists due to their potential for the development of new therapeutic agents. These compounds are known to modulate a complex network of processes, which involves regulatory biogenic purines. The purine-like scaffolds incorporating a 1,3,5-triazine ring became privileged in the construction of bioactive molecules [1]. It was found that pyrazolo[1,5-*a*][1,3,5]triazine (5-aza-9-deazapurine) [2] and 1,2,4-triazolo[1,5-*a*][1,3,5]triazine (5-azapurine) [3,4] were the most promising skeletons in this group of purine isosteres. We developed efficient methods for the synthesis of adenine and xanthine analogues built on these scaffolds [5,6,7,8,9,10,11,12,13,14,15]. The synthesis of 5-aza-guanines has also been reported [16,17]. 

Isoguanine has been reported as a skeleton for several biologically important alkaloids [18,19,20]. Some interesting results were obtained on a compound with the 4-aminopyrazolo[1,5-*a*][1,3,5]triazin-2-one skeleton (a triazine-based 5-aza-9-deaza-isostere of isoguanine) [21]. This compound was an efficient inhibitor of purine nucleoside phosphorylase, which is a valid target for anticancer and antiparasitic therapy [22,23,24,25]. This brought our attention to a similar purine-like heterocyclic system, i.e., 5-aza-isoguanine. Herein, we report our attempts to develop an efficient synthesis of 5-aza-isoguanines bearing various substituents on the amino group. 

## 2. Results and Discussion 

**Scheme 1:**
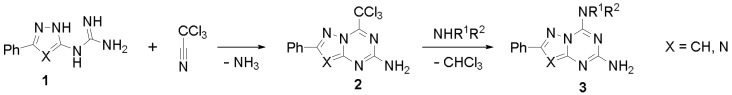
Synthesis of diamino-substituted azolo[1,5-*a*][1,3,5]triazines **3**.

Earlier we developed convenient syntheses of amino-substituted pyrazolo[1,5-*a*][1,3,5]triazines and 1,2,4-triazolo[1,5-*a*][1,3,5]triazines exploiting good reactivity of highly electrophilic 1,3,5-triazine ring substituted with the trichloromethyl group [26,27]. The key step in this synthetic approach was the ring closure reaction of azolylguanidine **1** using trichloroacetonitrile (Scheme 1). The subsequent nucleophilic substitution of the introduced trichloromethyl leaving group of **2** with variety of amines resulted in the formation of the corresponding pyrazolo[1,5-*a*][1,3,5]triazines and 1,2,4-triazolo[1,5-*a*][1,3,5]triazines **3**. 

**Scheme 2:**
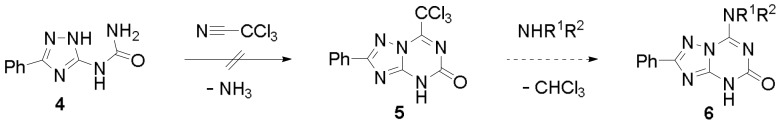
An attempt to prepare 5-aza-isoguanines via trichloromethyl-substituted intermediate **5**.

With our interest leaning towards the synthesis of 5-aza-isoguanines, which are 5-oxo-analogues of compounds **3**, an initial attempt to prepare 1,2,4-triazolo[1,5-*a*][1,3,5]triazine 5 bearing reactive trichloromethyl group was based on the previously developed reaction using triazolylurea **4** in place of guanidine **1** (Scheme 2). However, despite a number of attempts to achieve the triazine ring closure using trichloroacetonitrile under various reaction conditions, we were able to isolate starting urea **4** only. 

**Scheme 3:**
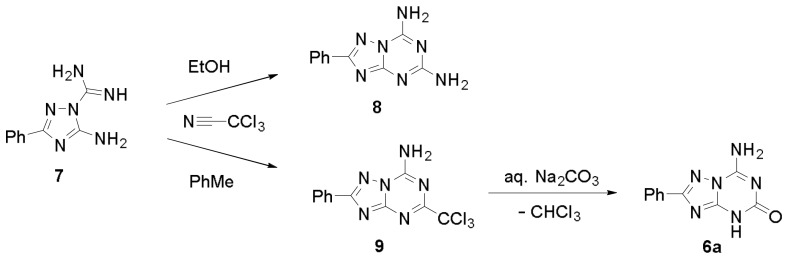
Synthesis of 5-aza-isoguanine **6a**.

To further explore the trichloromethyl chemistry in the synthesis of 7-amino-substituted 1,2,4-triazolo[1,5-*a*][1,3,5]triazin-5-ones **6**, we subjected 1-guanyl-1,2,4-triazole **7**, prepared as reported previously [28], to the reaction with trichloroacetonitrile (Scheme 3). Similar to triazolylguanidine **1** (X = N), the reaction pathway of **7** was solvent dependent. In ethanol, diamine **8**, identical to that reported in reference [29], was formed, whereas 1,2,4-triazolo[1,5-*a*][1,3,5]triazine **9** was isolated exclusively when the reaction was carried out in toluene. 

In the ^1^H-NMR spectrum of **9**, two protons of the amino group gave independent signals appearing at rather low field: 9.22 and 9.65 ppm. These observations can be attributed to the prominent delocalisation of an electron pair on the amino group nitrogen directly attached to the highly electron-deficient 1,3,5-triazine ring. The electron-withdrawing trichloromethyl group further decreased electron density on the triazine ring, enhancing the partial double bond character of the C-NH_2_ bond and restricting the rotation around it. The activation energy (ΔG^‡^) for the hindered rotation around this bond was estimated using dynamic ^1^H-NMR spectroscopy and was equal 68.8 kJ at the coalescence temperature 343 K. 

**Scheme 4:**
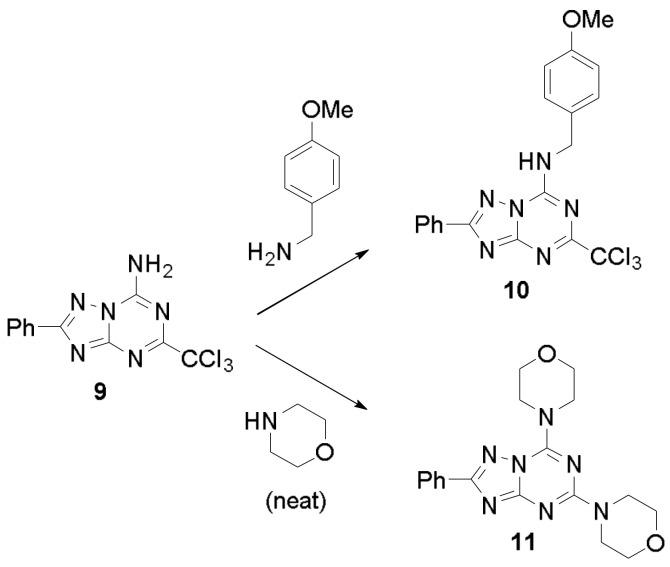
Reaction of **9** with 4-methoxybenzylamine and morpholine.

The trichloromethyl group of **9** was hydrolytically removed using aqueous solution of sodium carbonate to afford **6a**. The substitution of trichloromethyl group of **9** with nucleophiles (particularly amines) was more problematic in comparison to the aminolysis of the isomeric structure **2**. No reaction was observed when similar reaction conditions were applied. Moreover, unexpected results were obtained after prolonged heating with excess of amine depending on the reaction conditions and amine structure (Scheme 4). Replacement of the amino group in position 7 of the 1,2,4-triazolo[1,5-*a*][1,3,5]triazine ring was observed instead of the proposed substitution of the trichloromethyl group of 9 (e.g., in the synthesis of 10) or both the processes took place together (e.g., in the preparation of 11). It was demonstrated earlier that compounds with identical leaving groups in positions 5 and 7 of the 1,2,4-triazolo[1,5-*a*][1,3,5]triazine ring were suitable for the sequential nucleophilic substitution with amines: first in position 7, then in position 5 [30]. This strategy was successfully applied for the synthesis of bioactive 1,2,4-triazolo[1,5-*a*][1,3,5]triazines [31,32]. However, to the best of our knowledge, no examples on transaminations at the position 7 of 1,2,4-triazolo[1,5-*a*][1,3,5]triazines have been reported. For similar pyrazolo[1,5-*a*][1,3,5]triazines, the replacement of a N-methylanilino substituent in the corresponding position with other amines was effectively employed in drug-discovery programs to prepare a variety of compounds with a diverse substitution pattern [33,34,35,36,37,38,39,40]. 

**Scheme 5:**
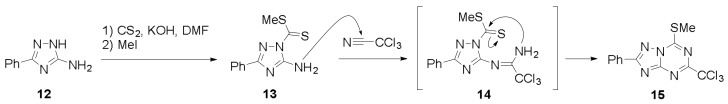
Synthesis of 5-trichloromethyl-7-methylthio-2-phenyl-1,2,4-triazolo[1,5-*a*][1,3,5]triazine (**15**).

The hydrolysis of 7-amino substituted **10** can be applied to generate **6k**. Nevertheless, difficulties with controlling reaction outcome in the synthesis of analogues of **10** and a long reaction time led us to design an intermediate with a leaving group more accessible for the nucleophilic substitution by amines than 9. This strategy was realized by the preparation of 1,2,4-triazolo[1,5-*a*][1,3,5]triazine (**15**) (Scheme 5). Aminotriazole **12** reacted with carbon disulfide in DMF in the presence of potassium hydroxide followed by the selective S-methylation affording **13**. Treatment of **13** with trichloroacetonitrile resulted in the formation of **15**. Trichloroacetonitrile provided a C–N fragment for the construction of the triazine ring. We propose that the reaction involved an initial addition to the triple bond of trichloroacetonitrile followed by the intramolecular ring closure of intermediate **14** via nucleophilic substitution at the methyl dithiocarboxylate moiety. Surprisingly, hydrogen sulfide instead of methylthiol played the role of a leaving group in this reaction. The methylthio group of **15** can be readily replaced by stoichiometric quantity of amines, e.g., 4-methoxybenzylamine, providing **10**. However, a long reaction time at the hydrolysis step arising from the low aqueous solubility of **10** and the necessity of chromatographic purification limited the practicality of this approach. 

**Scheme 6:**
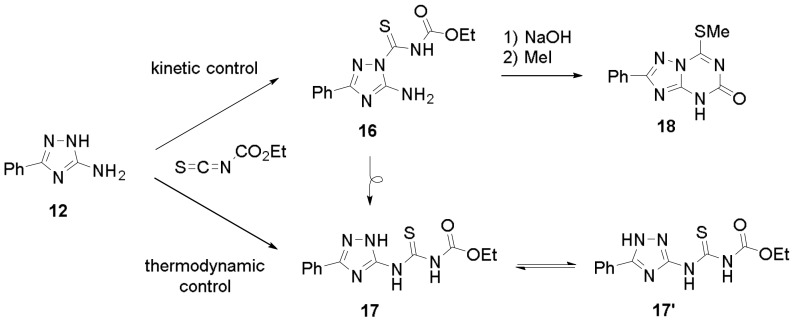
Synthesis of 7-methylthio-2-phenyl-1,2,4-triazolo[1,5-*a*][1,3,5]triazin-5-one (**18**).

**Figure 1:**
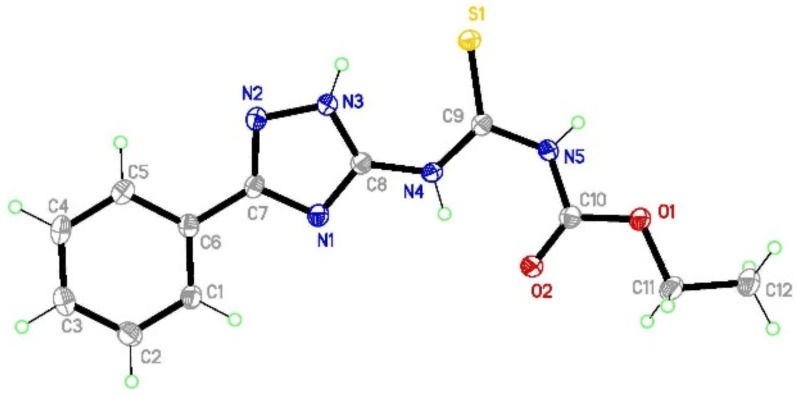
X-ray structure of **17**.

Considering the smooth replacement of the methylthio group at position 7 of the 1,2,4-triazolo[1,5-*a*][1,3,5]triazine with amines, we implemented an alternative synthetic approach for the preparation of **6**. The key intermediate in this protocol was **16**, prepared from 5-amino-3-phenyl-1,2,4-triazole (**12**) (Scheme 6). The selective addition of **12** to ethoxycarbonyl isothiocyanate was challenging due to the presence of several competing nucleophilic centers on **12**. It was found that regioselective addition occurs only at the endocyclic N-1 atom of **16** when the reaction was carried out under kinetic control in cold acetone for not more than 15 min. Extension of the reaction time or increasing temperature led to the formation of more thermodynamically stable *N*-carbethoxy-*N′*-(1,2,4-triazol-5-yl)thiourea, which existed in solution as a mixture of two tautomers **17** and **17′** (*K*_T_ = 0.74) crystalizing in the form of the predominant tautomer **17** (Figure 1) [41]. 

In solutions, **16** readily underwent rearrangement to **17** even at room temperature. Therefore, instant preparation of sufficiently pure **16** was critical, as changes in the product structure during purification were unavoidable. The alkali-induced cyclocondensation of 16 followed by S‑methylation afforded 7-methylthio-2-phenyl-1,2,4-triazolo[1,5-*a*][1,3,5]triazin-5-one (**18**). 

**Scheme 7:**
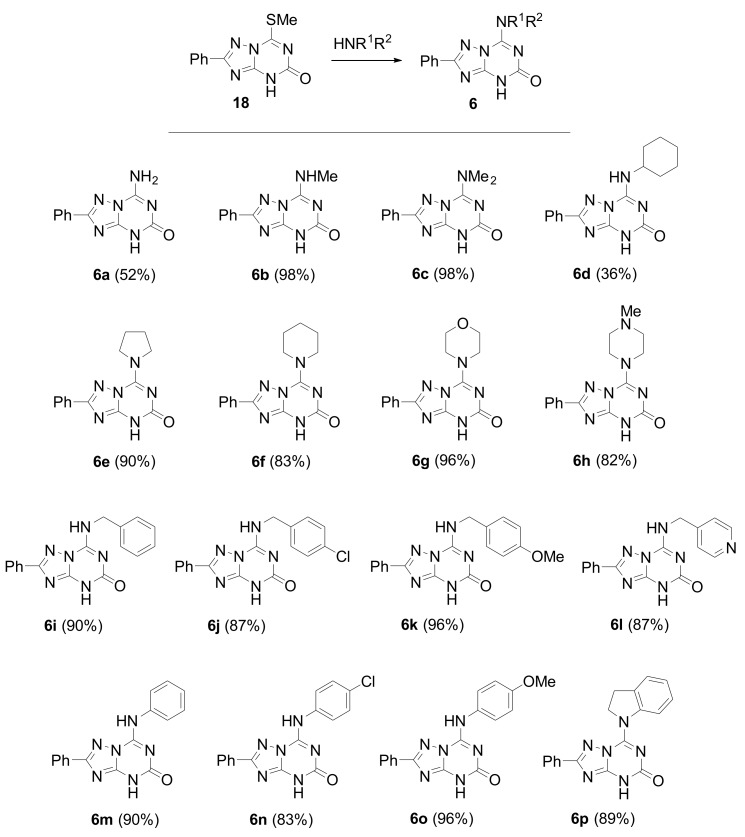
Synthesis of 5-aza-isoguanines **6**.

**Figure 2:**
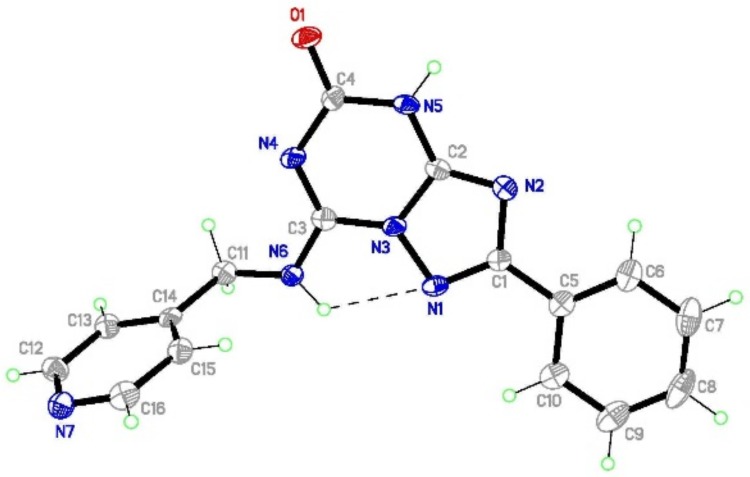
X-ray structure of **6l**.

The nucleophilic replacement of the methylthio group of **18** proceeded smoothly, with a variety of amines affording **6** with reasonable yields and high purity (Scheme 7). The reaction remained chemoselective even when aqueous solutions of fairly basic amines (synthesis of **6a**–**c**) were used. The structure of the prepared compounds **6** was confirmed by NMR spectroscopy, clearly showing that the signal of methylthio group of **18** was replaced by signals of the corresponding amines in spectra of **6**. The X-ray data for a representative example **6l** also confirmed the proposed structure (Figure 2) [42]. 

The synthetic route described in Scheme 6 and Scheme 7 was not placed on the top priority at the initial design of the synthetic routes due to the relatively low yield of the intermediate **16** and the uncertainty involved in the preparation of a pure regioisomer **16** as a result of the addition reaction between ethoxycarbonyl isothiocyanate to 5-amino-3-phenyl-1,2,4-triazole (**12**). However, this synthetic route unexpectedly became the most practical and versatile method that could be applied to the synthesis of a library of 7-amino substituted 2-phenyl-1,2,4-triazolo[1,5-*a*][1,3,5]triazin-5-ones (**6a**–**p**).

## 3. Conclusions 

In conclusion, several synthetic approaches to the synthesis of 5-aza-isoguanines **6** were explored to develop a practical method for their preparation. The significant difference in the reactivity of the C-5 and C-7 electrophilic centers of 1,2,4-triazolo[1,5-*a*][1,3,5]triazines towards nucleophiles was demonstrated. The most practical and general method for the preparation of 5-aza-isoguanines **6** involved a regioselective addition of 5-amino-3-phenyl-1,2,4-triazole (**12**) to ethoxycarbonyl isothiocyanate followed by an intramolecular ring closure and S-methylation to afford **18**, which could be effectively aminated with various amines. The prepared compound **6** resemble a known purine nucleoside phosphorylase inhibitor and could be interesting for further investigations as potential anticancer agents. 

## 4. Materials and Methods 

### 4.1. General

The melting points (uncorrected) were determined via the use of the Gallenkamp melting point apparatus. The ^1^H NMR and ^13^C NMR spectra were recorded on a Bruker DPX-300 spectrometer (Fällanden, Switzerland), using DMSO-*d*_6_ as a solvent and TMS as the internal reference. All the reactions were monitored with the use of the analytical TLC carried out on aluminum plates coated on Silica gel 60 F_254_ (Merck, Darmstadt, Germany) with detection by UV light.

### 4.2. Synthesis 

#### 4.2.1. Synthesis of 2-Phenyl-1,2,4-triazolo[1,5-*a*][1,3,5]triazin-5,7-diamine **8**

1-Guanyl-3-phenyl-1,2,4-triazol-5-amine (**7**, 0.3 g, 1.5 mmol) and trichloroacetonitrile (0.45 mL, 4.5 mmol) were heated in EtOH (10 mL) under reflux for 6 h. After cooling, the precipitated colorless product was filtered and washed with cold EtOH to give the product, which was found to be identical to compound **8** prepared earlier via an alternative method [29]. Yield 210 mg, 62%. 

#### 4.2.2. Synthesis of 7-Amino-2-phenyl-5-trichloromethyl-1,2,4-triazolo[1,5-*a*][1,3,5]triazine **9**

1-Guanyl-3-phenyl-1,2,4-triazol-5-amine (**7**, 0.3 g, 1.5 mmol) and trichloroacetonitrile (0.45 mL, 4.5 mmol) were refluxed in toluene (10 mL) for 6 h. After cooling, the colorless product was filtered and recrystalized from EtOH. Yield 310 mg, 63%; mp > 300 °C (EtOH). ^1^H-NMR (300 MHz, DMSO-*d*_6_): δ 7.53–7.65 (3H, m, H-3′, H-4′ and H-5′), 8.19–8.28 (2H, m, H-2′ and H-6′), 9.22 (1H, s, NH), 9.65 (1H, s, NH). ^13^C-NMR (75 MHz, DMSO-*d*_6_): δ 96.2, 126.9 (2C), 128.9 (2C), 129.7, 130.9, 151.6, 157.2, 164.1, 164.6. Combustion elemental analysis calculated for C_11_H_7_Cl_3_N_6_: C, 40.09; H, 2.14; N, 25.50. Found: C, 39.96; H, 2.35; N, 25.32. 

#### 4.2.3. Synthesis of 7-Amino-2-phenyl-1,2,4-triazolo[1,5-*a*][1,3,5]triazin-5-ones **6a** from **9**

To 10% aqueous Na_2_CO_3_ (10 mL), 7-amino-2-phenyl-5-trichloromethyl-1,2,4-triazolo[1,5-*a*][1,3,5]triazine (**9**, 0.33 g, 1 mmol) was added, and the mixture was heated under reflux for 72 h. The reaction mixture was allowed to cool and HCl (1 N) was added dropwise to pH 4. The precipitate formed was filtered and washed with water to give colorless crystalline powder. Yield 36 mg, 16%; mp > 300 °C (MeOCH_2_CH_2_OH). ^1^H-NMR (300 MHz, DMSO-*d*_6_): δ 7.48–7.58 (3H, m, H-3′, H-4′ and H-5′), 8.01–8.11 (2H, m, H-2′ and H-6′), 8.26 (1H, s, NH), 8.64 (1H, brs, NH), 12.17 (1H, brs, N(4)H). ^13^C-NMR (75 MHz, DMSO-d_6_): δ 126.5 (2C), 128.8 (2C), 129.6, 130.4, 149.8, 153.1, 153.6 (2C), 161.4. Combustion elemental analysis for C_10_H_8_N_6_O: C, 52.63; H, 3.53; N, 36.83. Found: C, 52.42; H, 3.71; N, 36.66.

#### 4.2.4. Synthesis of *N*-(4-Methoxybenzyl)-2-phenyl-5-trichloromethyl-1,2,4-triazolo[1,5-*a*][1,3,5]triazin-7-amine **10**

To the suspension of **9** (0.33 g, 1 mmol) in EtOH (5 mL), 4-methoxybenzylamine (0.36 mL, 3 mmol) was added and the mixture was heated under reflux for 24 h. After cooling, the resulting precipitate was filtered, washed with cold EtOH, dried and recrystallized from EtOH. Yield 212 mg, 47%; mp 174–175 °C (EtOH). ^1^H-NMR (300 MHz, DMSO-*d*_6_): δ 3.73 (3H, s, OMe), 4.73 (2H, d, *J* = 5.8 Hz, CH_2_), 6.91 (2H, d, *J* = 8.7 Hz, C-3′′ and C-5′′), 7.47 (2H, d, *J* = 8.7 Hz, C-2′′ and C-6′′), 7.52–7.61 (3H, m, H-3′, H-4′ and H-5′), 8.16–8.28 (2H, m, H-2′ and H-6′), 10.20 (1H, t, *J* = 5.8 Hz, NH). ^13^C-NMR (75 MHz, DMSO-*d*_6_): δ 43.7, 55.0, 96.1, 113.7 (2C′), 126.9 (2C), 128.9 (2C), 129.3, 129.7, 129.8 (2C), 130.9), 149.6, 157.1, 158.7, 163.9, 164.5. Combustion elemental analysis calculated for C_19_H_15_Cl_3_N_6_O: C, 50.74; H, 3.36; N, 18.69. Found: C, 50.55; H, 3.52; N, 18.42.

#### 4.2.5. Synthesis of 5,7-Bis(morpholino)-2-phenyl-1,2,4-triazolo[1,5-*a*][1,3,5]triazine **11**

To 5 mL of morpholine, **9** (0.33 g, 1 mmol) was added, and the mixture was heated under reflux for 5 h. After cooling, the resulting precipitate was filtered, washed with cold EtOH, dried and recrystallized from EtOH. Yield 180 mg, 49%; mp 278–279 °C (EtOH). ^1^H-NMR (300 MHz, DMSO-*d*_6_): δ 3.61–3.85 (12H, m), 4.27 (4H, br. s, W_1/2_ = 24.9 Hz), 7.46–7.55 (3H, m, H-3′, H-4′ and H-5′), 8.05–8.15 (2H, m, H-2′ and H-6′). ^13^C-NMR (75 MHz, DMSO-*d*_6_): δ 44.0 (br. s), 46.7, 65.7, 65.8, 126.6 (2C), 128.6 (2C), 130.2 (2C), 148.5, 158.8, 161.1, 162.1. Combustion elemental analysis calculated for C_18_H_21_N_7_O_2_: C, 58.84; H, 5.76; N, 26.69. Found: C, 58.61; H, 6.02; N, 26.41.

#### 4.2.6. Synthesis of Methyl 5-Amino-3-phenyl-1,2,4-triazoyl-1-dithiocarbonate **13**

3-Phenyl-1,2,4-triazol-5-amine (**12**, 3.20 g, 20 mmol) was dissolved in cold DMF (8 mL). Carbon disulphide (1.2 mL, 20 mmol) was added drop-wise, followed by 10 N KOH solution (2 mL, 20 mmol). The reaction mixture was stirred for 1 h on an ice-bath. Iodomethane (1.25 mL, 20 mmol) was added, and stirring was continued for 10 min. Subsequently, the ice-bath was removed and the reaction mixture was stirred for 2 h. Cold water (15 mL) was added to the mixture, the yellow precipitate was filtered, washed with water, dried and recrystalized from EtOH. Yield 80 mg, 32%; mp 207–209 °C (EtOH). ^1^H-NMR (300 MHz, DMSO-*d*_6_): δ 2.63 (3H, s, SMe), 7.47–7.57 (3H, m, H-3′, H-4′ and H-5′), 8.03 (2H, dd, *J* = 6.8, 3.0 Hz, H-2′ and H-6′), 8.64 (2H, s, NH_2_). ^13^C-NMR (75 MHz, DMSO-*d*_6_): δ 18.8, 126.6 (2C), 128.8 (2C), 129.1, 130.6, 157.5, 158.8, 199.5. Combustion elemental analysis calculated for C_10_H_10_N_4_S_2_: C, 47.98; H, 4.03; N, 22.38. Found: C, 48.06; H, 4.12; N, 22.14.

#### 4.2.7. Synthesis of 7-Methylthio-2-phenyl-5-trichloromethyl-1,2,4-triazolo[1,5-*a*][1,3,5]triazine **15**

Methyl 5-amino-3-phenyl-1,2,4-triazoyl-1-dithiocarbonate (**13**, 0.75 g, 3 mmol) and trichloroacetonitrile (0.75 mL, 7.5 mmol) were heated under reflux in toluene (10 mL) for 12 h. Solvent was evaporated under reduced pressure, the residue was triturated with diethyl ether and solid was collected by filtration. Yield 245 mg, 68%. ^1^H-NMR (300 MHz, DMSO-*d*_6_): δ 2.85 (3H, s, SMe), 7.56–7.68 (3H, m, H-3′, H-4′ and H-5′), 8.26 (2H, dd, *J* = 6.6, 2.8 Hz, H-2′ and H-6′). ^13^C-NMR (75 MHz, DMSO-d_6_): δ 13.1, 95.2, 127.2 (2C), 129.0, 129.1 (2C), 131.6, 155.2, 161.2, 164.4, 166.0. Combustion elemental analysis calculated for C_12_H_8_Cl_3_N_5_S: C, 39.97; H, 2.24; N, 19.42. Found: C, 40.12; H, 2.33; N, 19.11.

#### 4.2.8. Reactions of 3-Phenyl-1,2,4-triazol-5-amine (**12**) with Ethoxycarbonyl Isothiocyanate

Synthesis of 5-amino-1-carbethoxythiocarbamoyl-1,2,4-triazole (**16**). To a solution of acetone (30 mL), 3-phenyl-1,2,4-triazol-5-amine (**12**, 3.20 g, 20 mmol) and ethoxycarbonyl isothiocyanate (2.62 mL, 20 mmol) were added. The reaction mixture was stirred on an ice-bath for 15 min. The yellow precipitate formed was filtered immediately. Yield 2.16 g, 37%; mp 207–209 °C. ^1^H-NMR (300 MHz, DMSO-*d*_6_): δ 2.63 (3H, s, SMe), 7.47–7.57 (3H, m, H-3′, H-4′ and H-5′), 8.03 (2H, dd, *J* = 6.8, 3.0 Hz, H-2′ and H-6′), 8.64 (2H, s, NH_2_). ^13^C-NMR (75 MHz, DMSO-d_6_): δ 18.8, 126.6 (2C), 128.8 (2C), 129.1, 130.6, 157.5, 158.8, 199.5. Combustion elemental analysis calculated for C_12_H_13_N_5_O_2_S: C, 49.47; H, 4.50; N, 24.04. Found: C, 49.20; H, 4.67; N, 23.88. 

Synthesis of *N*-carbethoxy-*N′*-(3-phenyl-1H-1,2,4-triazol-5-yl)thiourea (**17**). To the solution of 3-phenyl-1,2,4-triazol-5-amine (**12**, 0.48 g, 3 mmol) in anhydrous DMF (4 mL), ethoxycarbonyl isothiocyanate (0.43 mL, 3.3 mmol) was added. After stirring the mixture for 5 h at room temperature, cold water (60 mL) was added. The precipitated product was filtered, washed with cold water and recrystalized from EtOH. Yield 0.74 g, 85%; mp 180–181 °C (EtOH). ^1^H-NMR (300 MHz, DMSO-d_6_): δ 1.28 (3H, t, *J* = 7.1 Hz, CH_3_), 4.25 (2H, q, *J* = 7.1 Hz, CH_2_), 7.37–7.66 (3H, m, H-3′, H-4′ and H-5′), 8.01 (2H, d, *J* = 7.2 Hz, H-2′ and H-6′), 11.56* (12H, brs, 2NH), 11.87 (1H, brs, NH), 12.16 (1H, brs, NH), 13.93 (1H, brs, N(1)H), 14.47 (1H, brs, N(1)H)*. *—signals of the minor tautomer. Combustion elemental analysis calculated for C_12_H_13_N_5_O_2_S: C, 49.47; H, 4.50; N, 24.04. Found: C, 49.33; H, 4.62; N, 23.95. 

#### 4.2.9. Synthesis of 7-Methylthio-2-phenyl-1,2,4-triazolo[1,5-*a*][1,3,5]triazin-5-one **18**

5-Amino-1-carbethoxythiocarbamoyl-1,2,4-triazole (**16**, 2.9 g, 10 mmol) was heated under reflux in aqueous EtOH (80%, 20 mL) for 20 min and then cooled. The creamy product was filtered, dissolved in 5 mL of water and then stirred with iodomethane (0.62 mL, 10 mmol) for 30 min at 10 °C with a slow warming to room temperature. The colorless precipitate formed was filtered and dissolved in water (30 mL). A solution of HCl (1 N) was then added drop-wise to pH 3. The colorless precipitate formed was filtered, washed with water and dried. Yield 1.68 g, 65%; mp 275–276 °C. ^1^H-NMR (300 MHz, DMSO-*d*_6_): δ 2.62 (3H, s, SMe), 7.49–7.59 (3H, m, H-3′, H-4′ and H-5′), 8.00–8.11 (2H, m, H-2′ and H-6′), 13.07 (1H, s, N(4)H). ^13^C-NMR (75 MHz, DMSO-d_6_): δ 12.8, 126.7 (2C), 128.9 (2C), 130.0, 130.8, 151.2, 152.1 (2C), 162.2, 162.6. Combustion elemental analysis calculated for C_11_H_9_N_5_OS: C, 50.96; H, 3.50; N, 27.01. Found: C, 50.77; H, 3.62; N, 26.90. 

#### 4.2.10. Synthesis of 7-Amino-2-phenyl-1,2,4-triazolo[1,5-*a*][1,3,5]triazin-5-one **6a** from **18**

7-Methylthio-2-phenyl-1,2,4-triazolo[1,5-*a*][1,3,5]triazin-5-one (**18**, 0.52 g, 2 mmol) was added to a solution of aqueous NH_3_ (28%, 20 mL), and the mixture was stirred on a water bath at 55 °C for 18 h. The colorless precipitate formed was filtered and recrystalized from MeOCH_2_CH_2_OH. Yield 238 mg, 52%. Analytical data obtained were identical to those of compound **6a** obtained from the method described under Section 4.2.3. 

#### 4.2.11. Synthesis of 7-Amino-substituted-2-phenyl-1,2,4-triazolo[1,5-*a*][1,3,5]triazin-5-ones **6b** and **6c**

7-Methylthio-2-phenyl-1,2,4-triazolo[1,5-*a*][1,3,5]triazin-5-one (**18**, 0.52 g, 2 mmol) was added to an aqueous solution of methylamine (40%) or dimethylamine (40%) (10 mL) and the mixture was stirred at room temperature for 12 h. Solvent was evaporated under reduced pressure and recrystalized from a suitable solvent. 

*7-Methylamino-2-phenyl-1,2,4-triazolo[1,5-a][1,3,5]triazine-5-one* (**6b**). Colorless crystalline powder; yield 474 mg, 98%; mp 298–299 °C (EtOH); ^1^H-NMR (300 MHz, DMSO-*d*_6_): δ 2.94 (3H, d, *J* = 3.8 Hz, NHMe), 7.48–7.58 (3H, m, H-3′, H-4′ and H-5′), 8.02–8.11 (2H, m, H-2′ and H-6′), 8.71 (1H, q, *J* = 3.8 Hz, NH), 12.18 (1H, s, N(4)H). ^13^C-NMR (75 MHz, DMSO-*d*_6_): δ 27.3, 126.5 (2C), 128.8 (2C), 129.6, 130.4, 148.3, 152.8, 153.4 (brs), 161.3. Combustion elemental analysis calculated for C_11_H_10_N_6_O: C, 54.54; H, 4.16; N, 34.69. Found: C, 54.46; H, 4.21; N, 34.60. 

*7-Dimethylamino-2-phenyl-1,2,4-triazolo[1,5-a][1,3,5]triazine-5-one* (**6c**). Colorless crystalline powder; yield 500 mg, 98%; mp > 300 °C (MeOCH_2_CH_2_OH); ^1^H-NMR (300 MHz, DMSO-*d*_6_): δ 3.47 (6H, brs, NMe_2_), 7.49–7.57 (3H, m, H-3′, H-4′ and H-5′), 8.00–8.10 (2H, m, H-2′ and H-6′), 12.24 (1H, s, N(4)H). Combustion elemental analysis calculated for C_12_H_12_N_6_O: C, 56.24; H, 4.72; N, 32.79. Found: C, 56.11; H, 4.89; N, 32.62. 

#### 4.2.12. Synthesis of 7-Amino-substituted-2-phenyl-1,2,4-triazolo[1,5-*a*][1,3,5]triazin-5-ones **6d**–**6p**.

7-Methylthio-2-phenyl-1,2,4-triazolo[1,5-*a*][1,3,5]triazin-5-one (18, 0.52 g, 2 mmol) was added to a solution of appropriate amine (3 mmol) in DMF (5 mL), and the mixture was heated at 70–80 °C with stirring for 4–14 h. After cooling, ice cold water (40 mL) was added and the product was filtered and recrystalized from a suitable solvent.

*7-Cyclohexylamino-2-phenyl-1,2,4-triazolo[1,5-a][1,3,5]triazin-5-one* (**6d**). Colorless crystalline powder; yield 245 mg, 36%; mp > 300 °C (EtOH). ^1^H-NMR (300 MHz, DMSO-*d*_6_): δ 1.03–1.21 (1H, m, H_c-Hex_), 1.23-1.42 (2H, m, H_c-Hex_), 1.44–1.69 (3H, m, H_c-Hex_), 1.71–1.92 (4H, m, H_c-Hex_), 3.85–4.01 (1H, m, H-1′′), 7.47–7.59 (3H, m, H-3′, H-4′ and H-5′), 8.02–8.16 (2H, m, H-2′ and H-6′), 8.56 (1H, d, *J* = 8.3 Hz, NH), 12.17 (1H, s, N(4)H). ^13^C-NMR (75 MHz, DMSO-*d*_6_): δ 24.85 (2C), 24.89, 31.5 (2C), 50.0, 126.6 (2C), 128.7 (2C), 129.6, 130.4, 147.1, 152.9, 153.5 (2C), 161.3. Combustion elemental analysis calculated for C_16_H_18_N_6_O: C, 61.92; H, 5.85; N, 27.08. Found: C, 61.86; H, 6.02; N, 26.89.

*2-Phenyl-7-pyrrolidino-1,2,4-triazolo[1,5-a][1,3,5]triazin-5-one* (**6e**). Colorless crystalline powder; yield 508 mg, 90%; mp > 300 °C (DMSO); ^1^H-NMR (300 MHz, DMSO-*d*_6_): δ 1.82–2.08 (4H, m, C(3′′)H_2_C(4′′)H_2_), 3.64 (1H, t, *J* = 6.0 Hz, C(5′′)H_2_), 4.25 (1H, t, *J* = 6.0 Hz, C(2′′)H_2_), 7.48–7.57 (3H, m, H-3′, H-4′ and H-5′), 8.00–8.09 (2H, m, H-2′ and H-6′), 12.15 (1H, s, N(4)H). Combustion elemental analysis calculated for C_14_H_14_N_6_O: C, 59.56; H, 5.00; N, 29.77. Found: C, 59.43; H, 5.24; N, 29.51.

*7-Piperidino-2-phenyl-1,2,4-triazolo[1,5-a][1,3,5]triazin-5-one* (**6f**). Colorless crystalline powder; yield 492 mg, 83%; mp 286 °C (MeOCH_2_CH_2_OH). ^1^H-NMR (300 MHz, DMSO-*d*_6_): δ 1.57–1.78 (6H, m, C(3′′)H_2_, C(4′′)H_2_, C(5′′)H_2_, 3.80–4.50 (4H, m, C(2′′)H_2_, C(6′′)H_2_, 7.47–7.58 (3H, m, H-3′, H-4′ and H-5′), 7.98–8.10 (2H, m, H-2′ and H-6′), 12.29 (1H, s, N(4)H). ^13^C-NMR (75 MHz, DMSO-*d*_6_): δ 23.7, 25.5, 47.9, 126.5 (2C), 128.8 (2C), 129.3, 130.5, 147.4, 152.8, 154.9 (2C), 160.4. Combustion elemental analysis calculated for C_15_H_16_N_6_O: C, 60.80; H, 5.44; N, 28.36. Found: C, 60.68; H, 5.63; N, 28.21.

*7-Morpholino-2-phenyl-1,2,4-triazolo[1,5-a][1,3,5]triazin-5-one* (**6g**). Colorless crystalline powder; yield 573 mg, 96%; mp > 300 °C (MeOCH_2_CH_2_OH); ^1^H-NMR (300 MHz, DMSO-*d*_6_): δ 3.77 (4H, t, *J* = 4.5 Hz, CH_2_OCH_2_), 4.23 (4H, brs, CH_2_NCH_2_), 7.47–7.57 (3H, m, H-3′, H-4′ and H-5′), 8.00–8.10 (2H, m, H-2′ and H-6′), 12.39 (1H, s, N(4)H). ^13^C-NMR (75 MHz, DMSO-*d*_6_): δ 47.2 (brs), 65.7, 126.6 (2C), 128.8 (2C), 129.1, 130.6, 147.7, 152.7, 154.9 (2C), 160.5. Combustion elemental analysis calculated for C_14_H_14_N_6_O_2_: C, 56.37; H, 4.73; N, 28.17. Found: C, 56.22; H, 4.87; N, 28.02.

*7-N-methylpiperazino-2-phenyl-1,2,4-triazolo[1,5-a][1,3,5]triazin-5-one* (**6h**). Colorless crystalline powder; yield 510 mg, 82%; mp 266–268 °C (MeOCH_2_CH_2_OH); ^1^H-NMR (300 MHz, DMSO-*d*_6_): δ 2.24 (3H, s, NMe), 2.44–2.55 (4H, m, CH_2_N(Me)CH_2_), 4.24 (4H, brs, CH_2_NCH_2_), 7.47–7.59 (3H, m, H-3′, H-4′ and H-5′), 7.99–8.10 (2H, m, H-2′ and H-6′), 12.11 (1H, brs, N(4)H). ^13^C-NMR (75 MHz, DMSO-*d*_6_): δ 45.3, 46.6 (brs), 54.1, 126.5 (2C), 128.8 (2C), 129.2, 130.6, 147.6, 152.8, 154.9 (2C), 160.5. Combustion elemental analysis calculated for C_15_H_17_N_7_O: C, 57.87; H, 5.50; N, 31.49. Found: C, 57.64; H, 5.62; N, 31.35.

*7-Benzylamino-2-phenyl-1,2,4-triazolo[1,5-a][1,3,5]triazin-5-one* (**6i**). Colorless crystalline powder; yield 572 mg, 90%; mp > 300 °C (DMF); ^1^H-NMR (300 MHz, DMSO-*d*_6_): δ 4.63 (2H, d, *J* = 6.0 Hz, CH_2_), 7.27 (1H, t, *J* = 7.0 Hz, H-4′′), 7.35 (2H, t, *J* = 7.3 Hz, H-3′′ and H-5′′), 7.41 (2H, d, *J* = 7.9 Hz, H-2′′ and H-6′′), 7.48–7.59 (3H, m, H-3′, H-4′ and H-5′), 8.03–8.13 (2H, m, H-2′ and H-6′), 9.31 (1H, t, *J* = 6.0 Hz, NH), 12.24 (1H, s, N(4)H). ^13^C-NMR (75 MHz, DMSO-*d*_6_): δ 43.4, 126.5 (2C), 127.0, 127.4 (2C), 128.2 (2C), 128.8 (2C), 129.6, 130.5, 138.0, 148.2, 152.9, 153.5 (2C), 161.4. Combustion elemental analysis calculated for C_17_H_14_N_6_O: C, 64.14; H, 4.43; N, 26.40. Found: C, 63.89; H, 4.64; N, 26.19.

*7-(4-Chlorobenzylamino)-2-phenyl-1,2,4-triazolo[1,5-a][1,3,5]triazin-5-one* (**6j**). Colorless crystalline powder; yield 614 mg, 87%; mp > 300 °C (DMF); ^1^H-NMR (300 MHz, DMSO-*d*_6_): δ 4.61 (2H, d, *J* = 5.7 Hz, CH_2_), 7.40 (2H, d, *J* = 8.7 Hz, H-3′′ and H-5′′), 7.44 (2H, d, *J* = 8.7 Hz, H-2′′ and H-6′′), 7.48–7.59 (3H, m, H-3′, H-4′ and H-5′), 8.03–8.13 (2H, m, H-2′ and H-6′), 9.32 (1H, t, *J* = 5.7 Hz, NH), 12.25 (1H, s, N(4)H). ^13^C-NMR (75 MHz, DMSO-*d*_6_): δ 42.8, 126.5 (2C), 128.1 (2C), 128.8 (2C), 129.3 (2C), 129.5, 130.5, 131.6, 137.0, 148.2, 152.9, 153.4 (2C), 161.5. Combustion elemental analysis calculated for C_17_H_13_ClN_6_O: C, 57.88; H, 3.71; N, 23.82. Found: C, 57.71; H, 4.00; N, 23.69.

*7-(4-Methoxybenzylamino)-2-phenyl-1,2,4-triazolo[1,5-a][1,3,5]triazin-5-one* (**6k**). Colorless crystalline powder; yield 670 mg, 96%; mp > 300 °C (MeOCH_2_CH_2_OH); ^1^H-NMR (300 MHz, DMSO-*d*_6_): δ 3.73 (3H, s, OMe), 4.56 (2H, d, *J* = 6.0 Hz, CH_2_), 6.90 (2H, d, *J* = 8.7 Hz, H-3′′ and H-5′′), 7.35 (2H, d, *J* = 8.7 Hz, H-2′′ and H-6′′), 7.48–7.58 (3H, m, H-3′, H-4′ and H-5′), 8.03–8.12 (2H, m, H-2′ and H-6′), 9.25 (1H, t, *J* = 6.0 Hz, NH), 12.25 (1H, s, N(4)H). ^13^C-NMR (75 MHz, DMSO-*d*_6_): δ 42.9, 55.0, 113.6 (2C), 126.5 (2C), 128.8 (2C), 128.9 (2C), 129.5, 129.9, 130.4, 148.0, 152.9, 153.5 (brs), 158.4, 161.4. Combustion elemental analysis calculated for C_18_H_16_N_6_O_2_: C, 62.06; H, 4.63; N, 24.12. Found: C, 61.96; H, 4.88; N, 23.93.

*2-Phenyl-7-[(pyridin-4-ylmethyl)amino]-1,2,4-triazolo[1,5-a][1,3,5]triazin-5-one* (**6l**). Colorless crystalline powder; yield 556 mg, 87%; mp 293–294 °C (EtOH); ^1^H-NMR (300 MHz, DMSO-*d*_6_): δ 4.66 (2H, d, *J* = 5.7 Hz, CH_2_), 7.40 (2H, d, *J* = 4.9 Hz, H-3′′ and H-5′′), 7.48–7.61 (3H, m, H-3′, H-4′ and H-5′), 8.02-8.15 (2H, m, H-2′ and H-6′), 8.52 (2H, d, *J* = 4.9 Hz, H-2′′ and H-6′′), 9.36 (1H, t, *J* = 5.7 Hz, NH), 12.29 (1H, s, N(4)H). ^13^C-NMR (75 MHz, DMSO-*d*_6_): δ 42.5, 122.1 (2C), 126.5 (2C), 128.8 (2C), 129.5, 130.5, 146.9, 148.4, 149.4 (2C), 153.0, 153.4 (2C), 161.5. Combustion elemental analysis calculated for C_16_H_13_N_7_O: C, 60.18; H, 4.10; N, 30.70. Found: C, 59.97; H, 4.34; N, 30.51.

*2-Phenyl-7-phenylamino-1,2,4-triazolo[1,5-a][1,3,5]triazin-5-one* (**6m**). Colorless crystalline powder; yield 545 mg, 90%; mp 298–299 °C (MeOCH_2_CH_2_OH); ^1^H-NMR (300 MHz, DMSO-*d*_6_): δ 7.23 (1H, t, *J* = 7.9 Hz, H-4′′), 7.43 (2H, t, *J* = 7.9 Hz, H-3′′ and H-5′′), 7.49–7.61 (3H, m, H-3′, H-4′ and H-5′), 7.80 (2H, d, *J* = 7.9 Hz, H-2′′ and H-4′′), 8.09–8.21 (2H, m, H-2′ and H-6′), 10.44 (1H, s, NH), 12.45 (1H, s, N(4)H). ^13^C-NMR (75 MHz, DMSO-*d*_6_): δ 123.3 (2C), 125.2, 126.7 (2C), 128.4 (2C), 128.8 (2C), 129.5, 130.5, 136.4, 146.5, 153.1, 153.3 (2C), 161.4. Combustion elemental analysis calculated for C_16_H_12_N_6_O: C, 63.15; H, 3.97; N, 27.62. Found: C, 62.99; H, 4.24; N, 27.41.

*7-(4-Chlorophenylamino)-2-phenyl-1,2,4-triazolo[1,5-a][1,3,5]triazin-5-one* (**6n**). Colorless crystalline powder; yield 562 mg, 83%; mp > 300 °C (MeOCH_2_CH_2_OH); ^1^H-NMR (300 MHz, DMSO-*d*_6_): δ 7.48 (2H, d, *J* = 8.7 Hz, H-3′′ and H-5′′), 7.52–7.61 (3H, m, H-3′, H-4′ and H-5′), 7.87 (2H, d, *J* = 8.7 Hz, H-2′′ and H-4′′), 8.08–8.20 (2H, m, H-2′ and H-6′), 10.55 (1H, s, NH), 12.49 (1H, s, N(4)H). ^13^C-NMR (75 MHz, DMSO-*d*_6_): δ 124.7 (2C), 126.7 (2C) 128.4 (2C), 128.8 (2C), 129.0, 129.5, 130.6, 135.5, 146.4, 153.1 (2C), 161.5. Combustion elemental analysis calculated for C_16_H_11_ClN_6_O: C, 56.73; H, 3.27; N, 24.81. Found: C, 56.62; H, 3.36; N, 24.70.

*7-(4-Methoxyphenylamino)-2-phenyl-1,2,4-triazolo[1,5-a][1,3,5]triazin-5-one* (**6o**). Colorless crystalline powder; yield 650 mg, 96%; mp 296–297 °C (MeOCH_2_CH_2_OH); ^1^H-NMR (300 MHz, DMSO-*d*_6_): δ 3.78 (3H, s, OMe), 6.99 (2H, d, *J* = 8.7 Hz, H-3′′ and H-5′′), 7.49–7.60 (3H, m, H-3′, H-4′ and H-5′), 7.65 (2H, d, *J* = 8.7 Hz, H-2′′ and H-4′′), 8.08–8.19 (2H, m, H-2′ and H-6′), 10.35 (1H, s, NH), 12.37 (1H, s, N(4)H). ^13^C-NMR (75 MHz, DMSO-*d*_6_): δ 55.2, 113.6 (2C), 125.0 (2C), 126.7 (2C), 128.8 (2C), 129.1, 129.5, 130.6, 146.6, 153.1, 153.3 (2C), 156.8, 161.4. Combustion elemental analysis calculated for C_17_H_14_N_6_O_2_: C, 61.07; H, 4.22; N, 25.14. Found: C, 60.87; H, 4.49; N, 24.98.

*7-Indolino-2-phenyl-1,2,4-triazolo[1,5-a][1,3,5]triazin-5-one* (**6p**). Colorless crystalline powder; yield 588 mg, 89%; mp 297 °C (MeOCH_2_CH_2_OH); ^1^H-NMR (300 MHz, DMSO-*d*_6_): δ 3.27 (2H, t, *J* = 8.3 Hz, C(3′′)H_2_), 4.91 (2H, d, *J* = 8.3 Hz, C(2′′)H_2_), 7.13 (1H, t, *J* = 7.3 Hz, H-5′′), 7.27 (1H, t, *J* = 7.7 Hz, H-6′′), 7.34 (1H, d, *J* = 7.2 Hz, H-5′′), 7.44–7.60 (3H, m, H-3′, H-4′ and H-5′), 8.00–8.15 (2H, m, H-2′ and H-6′), 8.40 (1H, d, *J* = 7.9 Hz, H-7′′), 12.48 (1H, s, N(4)H). ^13^C-NMR (75 MHz, DMSO-*d*_6_): δ 27.9, 52.3, 118.9, 124.7, 124.8, 126.5 (2C), 126.7, 128.8 (2C), 129.3, 130.5, 133.1, 141.7, 146.1, 153.0, 154.6 (2C), 160.5. Combustion elemental analysis calculated for C_18_H_14_N_6_O: C, 65.44; H, 4.27; N, 25.44. Found: C, 65.23; H, 4.54; N, 25.19.
